# Supplementary material for: Mechanisms governing the pioneering and redistribution capabilities of the non-classical pioneer PU.1
Source: Nat Commun. 2020 Jan 21;11:402. doi: 10.1038/s41467-019-13960-2 (PMC6972792; doi:10.1038/s41467-019-13960-2)
Supplement: Supplementary file 7 — Source data [file 41467_2019_13960_MOESM7_ESM.zip › Source_Data/FigureS3/FigureS3H_MotifScanOutput/homerResults/motif14.similar.html]

motif14

## Information for motif14

C
A
G
T
T
C
A
G
A
T
G
C
A
C
G
T
A
G
C
T
C
A
G
T
A
T
G
C
C
G
A
T
A
T
C
G
C
G
T
A
  
Reverse Opposite:  

G
C
A
T
T
A
G
C
G
C
T
A
T
A
C
G
G
T
C
A
T
C
G
A
T
G
C
A
T
A
C
G
A
G
T
C
G
T
C
A
  

|  |  |
| --- | --- |
| p-value: | 1e-62 |
| log p-value: | -1.443e+02 |
| Information Content per bp: | 1.610 |
| Number of Target Sequences with motif | 987.0 |
| Percentage of Target Sequences with motif | 25.53% |
| Number of Background Sequences with motif | 6570.2 |
| Percentage of Background Sequences with motif | 14.67% |
| Average Position of motif in Targets | 315.0 +/- 335.2bp |
| Average Position of motif in Background | 211.1 +/- 139.9bp |
| Strand Bias (log2 ratio + to - strand density) | 0.1 |
| Multiplicity (# of sites on avg that occur together) | 1.16 |
| Motif File: | file (matrix) reverse opposite |

### Similar de novo motifs found

|  |  |  |  |  |  |  |  |
| --- | --- | --- | --- | --- | --- | --- | --- |
| Rank | Match Score | Redundant Motif | P-value | log P-value | % of Targets | % of Background | Motif file |
| 1 | 0.844 | A G T C C T A G A T G C A G C T A G C T A C G T G T A C | 1e-34 | -80.003969 | 40.22% | 30.40% | motif file (matrix) |
